# A 6-hour light-dark cycle reduces photosynthesis and leaf greenness in spring wheat at stem elongation through nitrate accumulation

**DOI:** 10.3389/fpls.2025.1655271

**Published:** 2025-09-12

**Authors:** Helena Clauw, Hans Van de Put, Abderahman Sghaier, Trui Kerkaert, Pieter Vermeir, Kathy Steppe

**Affiliations:** ^1^ Laboratory of Plant Ecology, Department of Plants and Crops, Faculty of Bioscience Engineering, Ghent University, Gent, Belgium; ^2^ Laboratory for Chemical Analysis, Department of Green Chemistry and Technology, Faculty of Bioscience Engineering, Ghent University, Gent, Belgium

**Keywords:** *Triticum aestivum* (wheat), pre-anthesis development, nitrogen metabolism, stem elongation, source-sink, SPAD, photosynthesis, turgor-driven growth

## Abstract

**Introduction:**

The closed environments of space farming and vertical farming systems allow for the implementation of innovative short light-dark cycles. These cycles have the potential to accelerate plant development and synchronize the short light period with off-peak electricity prices, thereby maximizing profitability. Previous work showed that growing spring wheat (*Triticum aestivum* L.) under a 6h-6h light-dark cycle resulted in accelerated heading.

**Methods:**

The present study investigates pre-anthesis development under this photoperiod, with a focus on the stem elongation phase as critical period for yield determination. In addition, the effect of transitioning to a 14h-10h light-dark cycle, mimicking spring conditions in the field, at heading was examined. This was assessed across sequential growth chamber experiments in combination with variations in light intensity and CO_2_ concentration. Wheat phenology and leaf traits, including SPAD values, nitrogen and nitrate content and photosynthetic rates, were monitored.

**Results:**

SPAD values, and thus leaf greenness, declined significantly in leaves developing around the start of stem elongation, leading to reduced photosynthetic rates. In these leaves, nitrate accumulation was detected. SPAD values increased following the shift to a 14h-10h light-dark cycle at heading, coinciding with higher photosynthetic rates.

**Discussion:**

These findings underscore the importance of aligning photoperiod regimes with plant developmental stages to optimize wheat production in controlled environments.

## 1 Introduction

Wheat (*Triticum aestivum* L.) supplies about 20% of the calories and proteins in the human diet worldwide (Shiferaw et al., 2013). Because of its high nutritional value, it is an important crop for human nutrition on space missions (Page and Feller, 2013; Dong et al., 2014; Shewry and Hey, 2015). Also in the case of food system disruptions, and in extreme climates, growing staple crops like wheat in vertical farming systems has the potential to increase food security (Asseng et al., 2020). High capital and energy costs, however, limit the economic viability of these systems (Asseng et al., 2020; Eichelsbacher et al., 2025). In space farming and, by extension, vertical farming, a short growth cycle allows multiple harvests per year (Page and Feller, 2013; Asseng et al., 2020), significantly increasing the cumulative annual yield per ha and per year compared to the global average (Asseng et al., 2020). Under closed conditions, elevated CO_2_ concentrations can be implemented to support wheat yield (Monje and Bugbee, 1998). Further, by tailoring the light environment, including photoperiod and light intensity, we can accelerate development and enhance yield and quality (Clauw et al., 2024). As wheat is a long-day plant, a longer photoperiod accelerates development (Dong et al., 2014). However, the same outcome can be reached by shortening the dark period, which can be obtained by dividing the 24h cycle into several shorter cycles (Riddell et al., 1958). In addition, incorporating several night periods within a 24-hour cycle may allow turgor pressure to build up in each night period, potentially enhancing turgor-driven growth (Lockhart, 1965; Steppe et al., 2006, 2015; Coussement et al., 2021). In closed production systems, electricity for artificial lighting represents a significant cost (Asseng et al., 2020). Focusing on shorter nights rather than extended days offers a promising avenue to improve energy-efficiency. Moreover, installing a flexible short light period that aligns with low electricity price periods can substantially reduce the operational costs of indoor production systems (Avgoustaki and Xydis, 2021).

In earlier work, spring wheat was grown under a 6h-6h light-dark cycle, doubling the number of light-dark cycles per 24 hours using both a short day and night. Compared to a 12h-12h light-dark cycle with the same daily light integral and aligned with the Earth’s natural 24-hour rhythm, heading was accelerated while providing satisfactory grain yield and quality (Clauw et al., 2024). Additionally, it was shown that changing the 6h-6h light-dark cycle to a 14h-10h light-dark cycle around the onset of stem elongation, further improved grain development (Clauw et al., 2024). The 14h-10h light-dark cycle was chosen to mimic spring conditions in the field (Monostori et al., 2018), while only implementing two additional hours of light per 24 h compared to the 6h-6h light-dark cycle (12 hours of light in one 24 h cycle). While earlier work focused on accelerating the development, as well as grain yield and quality under a 6h-6h light-dark cycle, the physiological mechanisms underlying these findings remain unclear. Preliminary analysis from the same experiments revealed low SPAD values and nitrate accumulation in leaves developing around the stem elongation phase under this short photoperiod (Clauw et al., 2025). This phenomenon highlights the need to investigate the underlying mechanisms. To this end, the present study builds on these findings by further investigating measured key drivers of productivity, including leaf greenness, nitrogen status and photosynthesis.

**Figure 1:**
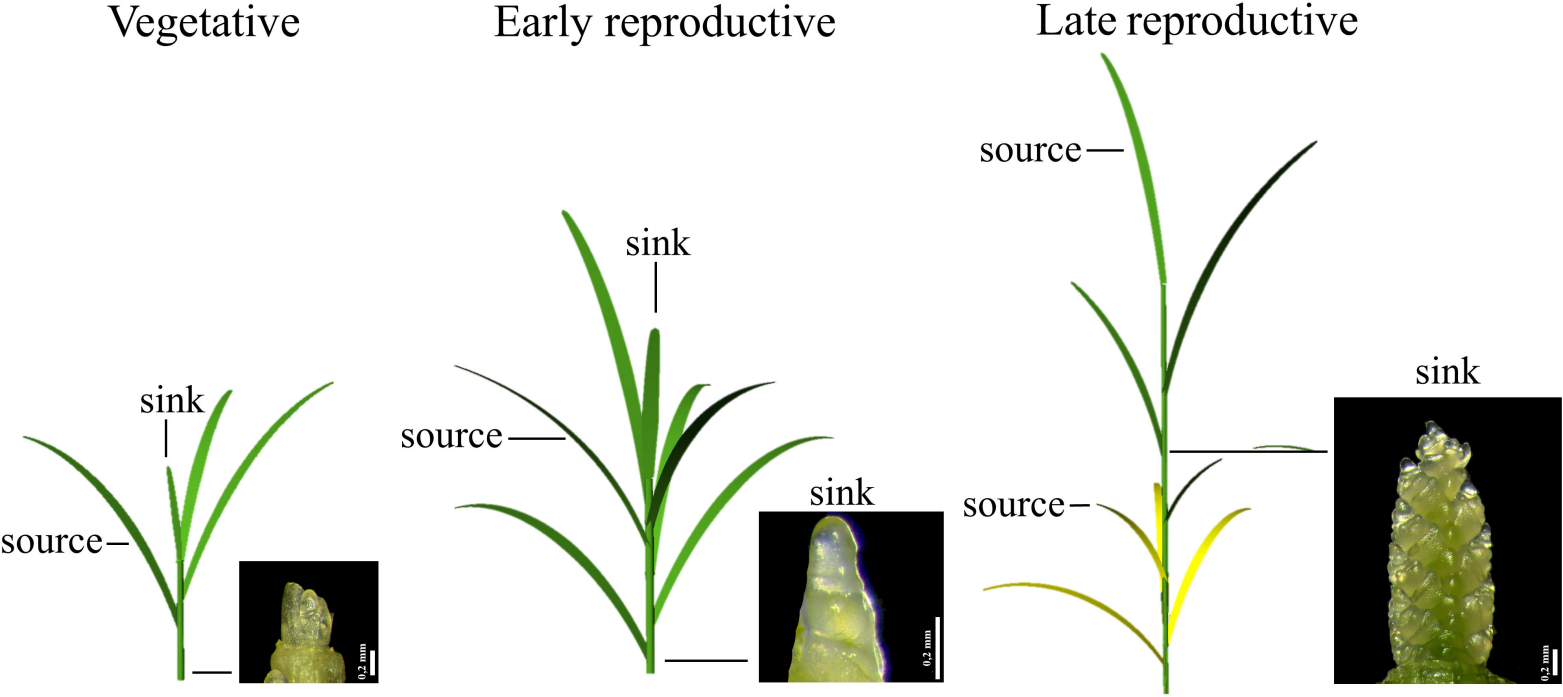
Schematic representation of wheat source-sink relationships during the phases of pre-anthesis development. Source and sink leaves as well as the apex at the specific phase are shown: vegetative phase (conical apex), early reproductive phase (double-ridge stage) and late reproductive phase (apex with floret primordia).

The photoperiod affects reproductive development of wheat by impacting the source-sink relationships and the nutrient supply to the reproductive structures (**Figure 1**; Gol et al., 2017). Initially, mature leaves are sucrose sources for developing leaves, and later during the development, also for the developing apex. During senescence, leaves are nitrogen (N) sources for the developing apex (Gol et al., 2017). Strong linear relationships between nitrogen and both Rubisco (ribulose-1,5-bisphosphate carboxylase/oxygenase) and chlorophyll have been shown (Evans, 1989). As Rubisco is the major N source for remobilization (Masclaux-Daubresse et al., 2010), this reduction results in the dismantling of the photosynthetic apparatus, and a decline in photosynthesis (Gregersen et al., 2008).

Wheat development until anthesis can be divided into the vegetative phase, during which leaf primordia are formed, the early reproductive phase, from the start of spikelet primordia development, until the formation of the terminal spikelet, and the late reproductive phase, from terminal spikelet initiation to flowering, during which the stem elongates and floret primordia develop into flowers (Slafer and Rawson, 1994; González et al., 2002; Gol et al., 2017). The third phase is critical for yield determination, making maximum light interception and photosynthesis essential during this period. During the late reproductive phase, the final number of florets, and consequently, the number of grains, is determined by floret death resulting from the competition for the limited assimilates between the elongating stem and the developing ear (Kirby, 1988; González et al., 2005).

As the source strength during stem elongation determines the final number of grains (Murchie et al., 2023), and both heading and stem elongation were accelerated under a 6h-6h light-dark cycle (Clauw et al., 2024), the current study aimed to investigate leaf characteristics during the pre-anthesis development of spring wheat under this photoperiod. The following questions were addressed: (1) does the early onset of stem elongation under a 6h-6h light-dark cycle, which is associated with accelerated heading, affect leaf blade physiology, (2) is pre-anthesis development improved by switching from a 6h-6h to a 14h-10h light-dark cycle at the onset of stem elongation, and (3) do higher light intensity and CO_2_ concentration support leaf blade physiology under a 6h-6h light-dark cycle? Our working hypotheses were: (1) the earlier onset of stem elongation will reduce blade growth, greenness, nitrogen content and photosynthesis due to competition between developing blades and the elongating stem; (2) transitioning to a longer 14h-10h light-dark cycle will alleviate these reductions; and (3) increased light intensity and CO_2_ concentration will stimulate leaf physiology under a 6h-6h light-dark cycle.

## 2 Materials and methods

### 2.1 Growth conditions

Two consecutive growth chamber experiments were conducted on spring wheat (*Triticum aestivum* L. cv. Servus) in two growth chambers (153 x 193 x 265 cm) at the Faculty of Bioscience Engineering at Ghent University, Belgium (51° 3′ N, 3° 42′ E). The growing system consisted of a tray holding 180 small baskets, with a 6 cm diameter and 7 cm height, that were filled with lava rock substrate and were submerged in a diluted Hoagland nutrient solution including NH_4_NO_3_ as nitrogen source (**Supplementary Table S1**). The tray measured 125 by 56 by 5 cm, resulting in a maximum volume of nutrient solution of 35 L. Twice a day, for five to ten minutes, fresh nutrient solution was added to the tray to compensate for evapotranspiration. Overflowing nutrient solution was captured at the opposite end of the tray. Before sowing, seeds were disinfected by immersing them in 70% ethanol for two minutes and in 20° household bleach for ten minutes, after which seeds were rinsed five times with deionized water. Three seeds were sown per basket. Later, the non-germinated and/or the surplus of germinated seeds were removed, resulting in one germinated seed per basket. This experimental design resulted in a plant density of 275 plants per m^2^. Of the 160 plants in total, the two outer rows, and four outer columns on the left and five on the right were assigned as border plants.

**Figure 2:**
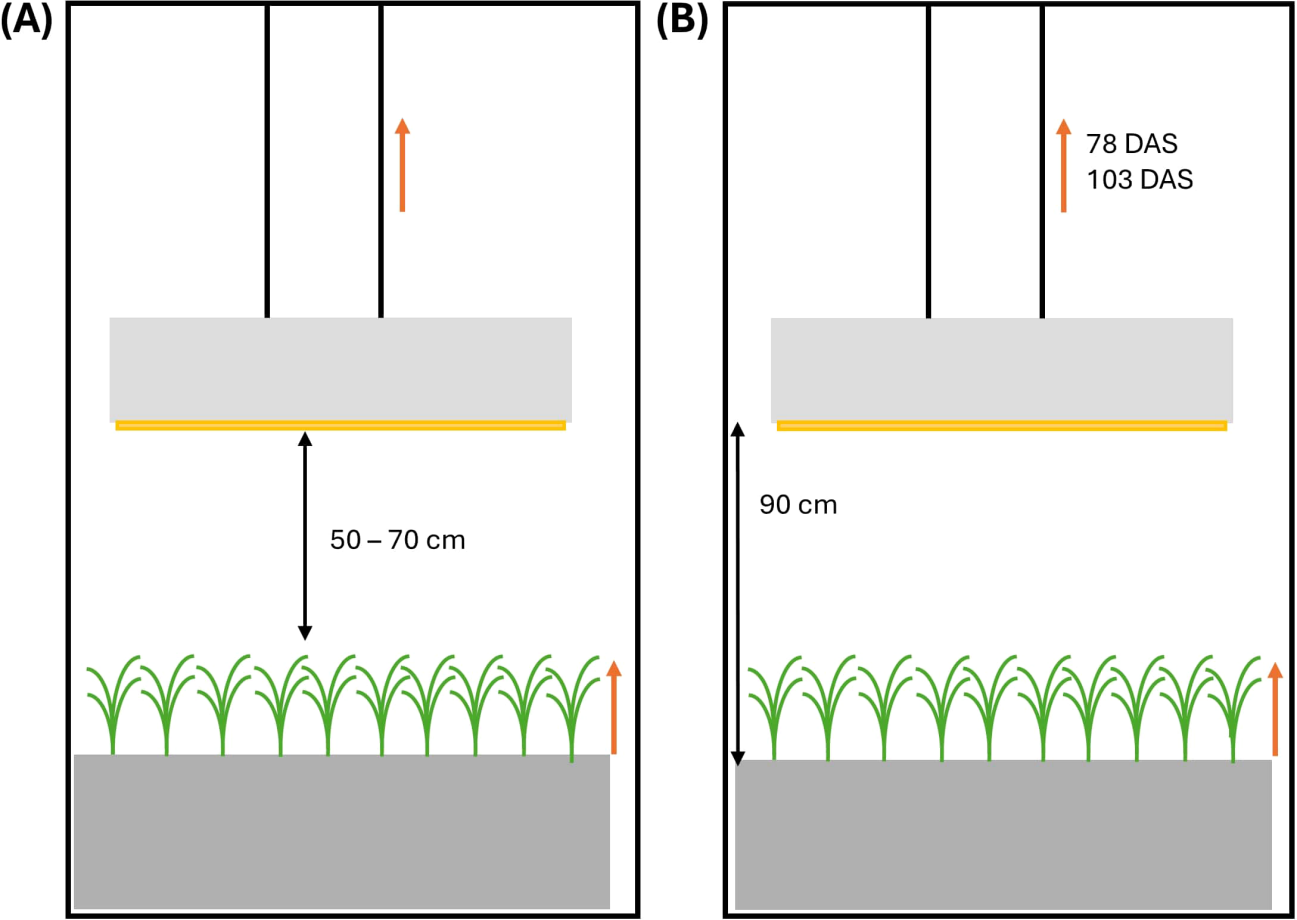
Schematic representation of the experimental setup in **(A)** the high light, elevated CO_2_ and dynamic (moveable) light fixtures experiment (HL + C_e_ + DY) and **(B)** the low light and ambient CO_2_ concentration experiment (LL + C_a_). Orange arrows indicate growth of the wheat plants or movement of the light fixtures. Movement was carried out on a regular basis, synchronized with plant growth, in the HL + C_e_ + DY experiment and on 78 and 103 days after sowing (DAS) in the LL + C_a_ experiment. Black double arrows indicate distances between the canopy and the light fixtures (HL + C_e_ + DY), or between tray and the light fixtures (LL + C_a_).

Plants were grown under a light intensity of 670 ± 5 µmol m^-2^ s^-1^ (pooled data from both growth chambers) measured at canopy level (high light intensity – HL) and at an average elevated CO_2_ concentration of 696 ± 73 ppm (elevated CO_2_ – C_e_). Five Alina lamps (RAYN Growing systems - ETC, London, UK) per growth chamber were installed to provide uniform lighting across the table. The light spectrum consisted of 12.3% blue, 32.0% green and yellow, 40.9% red and 14.8% far-red (**Supplementary Figure S1**). The lamps were raised regularly to maintain a distance of 50 to 70 cm above the canopy, resulting in a dynamic (DY) lamp height (**Figure 2A**). This experiment is referred to as HL + C_e_ + DY. Two different light-dark cycles were set: in the first chamber, a 6h-6h light-dark cycle was used until maturity, while in the second chamber, a 6h-6h light-dark cycle was used until the onset of stem elongation, when it was changed to a 14h-10h light-dark cycle. These treatments are referred to as the 6h-6h and 14h-10h treatments, respectively. The specific values of the environmental parameters, together with the codes for the experiments and treatments, are shown in **Table 1**. Light intensity at table level and ambient CO_2_ concentration were monitored by a LI-190 quantum sensor (LI-COR, Lincoln, Nebraska, US) and a GMP252 carbon dioxide probe (Vaisala CARBOCAP, Vantaa, Finland), respectively. Air temperature and relative humidity were measured with a temperature and relative humidity sensor (SHT25, Sensirion AG, Stäfa, Switzerland), installed in a ventilated radiation shield. In addition, nutrient solution dissolved oxygen (DO) concentration was measured with a portable pH/EC/DO meter (HI98199, Hanna Instruments, Temse, Belgium). Oxygen was actively added to the nutrient solution in the growth system through a venturi injector to maintain a suitable DO concentration. EC, pH and DO values of the nutrient solution on the table were measured every two to three days, directly before and after adding new nutrient solution to the system (**Supplementary Table S2**). The nutrient solution’s pH was lowered to 6.6 by adding nitric acid, and trays were flushed three times a week to keep EC, pH and DO constant.

**Table 1:** Environmental settings and variables with standard deviation for all light-dark cycles in the LL + C_a_ and HL + C_e_ + DY experiments: light intensity (µmol m^-2^ s^-1^); atmospheric CO_2_ concentration (ppm); light regime before and after the start of stem elongation; average measured electrical conductivity (EC, µS cm^-1^), pH and dissolved oxygen (DO, mg L^-1^) of the nutrient solution on the cultivation table; daytime air temperature (°C); nighttime air temperature (°C); relative humidity of the air (%).

Experiment code	LL + C_a_		HL + C_e_ + DY	
Treatment code	6h-6h	12h-12h	6h-6h	14h-10h
EXPERIMENT PARAMETERS				
Light intensity (µmol m^-2^ s^-1^)	93 ± 41^1^	93 ± 41^1^	658 ± 8²	682 ± 8²
CO_2_ concentration (ppm)	485 ± 97	473 ± 75	699 ± 72	693 ± 74
TREATMENT PARAMETERS				
Light-dark cycle before start stem elongation	6h-6h	12h-12h	6h-6h	6h-6h
Light-dark cycle after start stem elongation	6h-6h	12h-12h	6h-6h	14h-10h
OTHER ENVIRONMENTAL PARAMETERS				
EC (µS cm^-1^)	2142 ± 87	2169 ± 232	2010 ± 178	2030 ± 195
pH	7.2 ± 0.3	7.5 ± 0.3	6.6 ± 0.4	6.6 ± 0.3
DO (mg L^-1^)	NA	NA	3.50 ± 1.78	3.41 ± 1.69
Daytime temperature (°C)	20.0 ± 0.6	20.0 ± 0.7	20.6 ± 0.8	19.5 ± 1.9
Nighttime temperature (°C)	17.7 ± 0.7	17.8 ± 0.6	17.1 ± 0.8	18.0 ± 1.6
Relative humidity (%)	73.1 ± 10.0	73.1 ± 11.2	84.1 ± 7.9	81.2 ± 8.8

^1^Light intensity at table level; ^2^Light intensity at canopy level. Abbreviations: LL, ‘low light intensity’; HL, ‘high light intensity’; C_a_, ‘ambient CO_2_ concentration’; C_e_, ‘elevated CO_2_ concentration’; DY, ‘dynamic light fixture’.

In a second experiment, plants were grown under a low light intensity of 93 ± 41 µmol m^-2^ s^-1^ measured at table level (low light intensity – LL) and at an average ambient CO_2_ concentration of 479 ± 87 ppm (ambient CO_2_ – C_a_; pooled data from both growth chambers). The specific values of the environmental parameters, together with the codes for the experiments and treatments, are shown in **Table 1**. In the first growth chamber, a 12h-12h light-dark cycle was set, while in the second chamber, a 6h-6h light-dark cycle was set, both treatments having an equal daily light integral. These are referred to as the 12h-12h and 6h-6h treatments, respectively. Light was provided by prototype LED lamps (Rosa fixture, RAYN Growing systems - ETC, London, UK) at an initial distance of 90 cm above the tray (**Figure 2B**). The light spectrum consisted of 16.9% blue (400 - 500 nm), 14.6% green and yellow (500 – 600 nm), 54.6% red (600 – 700 nm) and 13.9% far-red (700 – 800 nm) (**Supplementary Figure S1**). The position of the lamps was static, but to deal with the plant’s elongation, lamps were raised to 100 cm above the table on 103 days after sowing (DAS) in the 6h-6h treatment, avoiding touching leaves and lamps. In the 12h-12h treatment, lamps were raised to 108 cm and 119 cm on 78 DAS and 103 DAS, respectively. This experiment is referred to as LL + C_a_. The dynamic setup used in the HL + C_e_ + DY experiment was developed as an optimized solution of this setup. Environmental parameters (**Table 1**) were continuously monitored with the same sensor set as in the HL + C_e_ + DY experiment. Nutrient solution pH and EC were regularly measured using a portable pH/EC/DO meter (HI98199, Hanna Instruments, Temse, Belgium).

### 2.2 Phenology and development

The onset of a certain phenological event was defined as an interval ending on the measurement day of first observation of this event and starting on the day after the previous measurement day. Differences in interval width between experiments may thus have resulted from differences in measurement frequency. Days after sowing (DAS) were converted to thermal time in growing degree days (°Cd; GDD) using sensor data of air temperature, measured every two minutes, assuming a base temperature of 0°C (Evers et al., 2005; González et al., 2005).

The onset of internode elongation was derived from destructive measurements on three plants per measurement day. In the HL + C_e_ + DY experiment, measurements were performed every 14 days on average, between 23 and 115 DAS. In the LL + C_a_ experiment, these measurements were performed every 20 days on average, between 20 and 133 DAS. The beginning of tillering and the end of leaf elongation on the main stem were derived from regular leaf length measurements, using a ruler, on the main stem and tillers of the same plants throughout the experiment. In the HL + C_e_ + DY experiment, blade length was measured every day, from three to seven DAS, every two to three days from seven to 27 DAS, and every three to four days from 27 to 59 DAS on four randomly selected plants per treatment. In the LL + C_a_ experiment, blade length was measured every day, from eight to 22 DAS, every two to three days from 22 to 46 DAS, every three to four days from 46 to 96 DAS and about every week from 96 to 116 DAS on six randomly selected plants per treatment. The beginning of tillering was defined as the day on which at least 50% of the measured plants had produced their first tiller(s).

### 2.3 Leaf characteristics and photosynthesis measurements

Blade greenness was measured on the same randomly selected plants used for leaf length measurements using a SPAD 502 Plus Chlorophyll Meter (Spectrum Technologies, Aurora, Illinois, United States). Three measurements were taken around the middle of the blade, avoiding the midrib (Debaeke et al., 2006), and then averaged. These measurements were performed at regular intervals to capture SPAD dynamics during the plants’ development. In the HL + C_e_ + DY experiment, SPAD values were measured on the same days and on the same leaves as blade length measurements, with two additional measurements on 62 and 67 DAS. In the LL + C_a_ experiment, measurements were taken approximately once a week on average.

Using a dataset of blade scans of the LL + C_a_ experiment, with a 6h-6h cycle, an average form factor of 0.78 was determined. This was calculated as the ratio between leaf area and the area of a rectangle of the same length and width (leaf area/(length*width); Dornbusch et al., 2009) for 51 leaves. It was assumed that this form factor was valid for both experiments, as no changes in morphology were observed.

Selecting other plants, length, width and SPAD of main stem blades were measured during destructive measurements. The plants that were removed from the table for destructive measurements were replaced by border plants of comparable dimensions to resemble the original shading conditions. Of the sampled blades, fresh weight (FW) and dry weight (DW), after oven-drying the samples at 70°C, were measured using a precision scale (ML104T/00, Mettler-Toledo Ltd., Melbourne, Australia). In the HL + C_e_ + DY experiment, leaf area was estimated by multiplying blade width and length with the average form factor. Using these data, DW per leaf area was calculated using the estimated leaf area and the measured DW for each leaf. The timing of the destructive measurements is described above (see ‘Phenology and development’).

In both experiments, gas exchange measurements were performed on one to three randomly selected plants per treatment using a LI-6400 portable photosynthesis system (LI-COR bioscience, Lincoln, Ne, USA). CO_2_ response curves (AC_i_) and light response curves (LRC) were acquired for the youngest to the third youngest leaves on the main stem of the measured plants at regular timesteps, resulting in a dataset spanning all leaf numbers. These measurements were performed about every week between 26 and 73 DAS for the HL + C_e_ + DY experiment. AC_i_ curves were measured on 29, 39, 71 and 73 DAS, and LRC curves were measured on 26, 38, 46 and 52 DAS. For the LL + C_a_ experiment these measurements were performed every week to two weeks between 13 and 90 DAS. AC_i_ curves were measured on 13 DAS, on the first leaf, and on 33 and 61 DAS, on the three youngest leaves. LRC curves were measured every week to two weeks on the three youngest leaves, except for 13 DAS, when only one leaf was developed. Air temperature and CO_2_ concentration in the gas exchange chamber were set equal to the values in the growth chamber. For AC_i_ curves, the light intensity for maximum photosynthesis was set to 1000 µmol m^-2^ s^-1^ in the LL + C_a_ experiment, and 1500 µmol m^-2^ s^-1^ in the HL + C_e_ + DY experiment.

### 2.4 Blade tissue nitrogen and nitrate analysis

Nitrogen (N) and nitrate (NO_3_^-^) content per unit of leaf area (mg m^-2^) were determined destructively for main stem and tiller blades with known SPAD values in the HL + C_e_ + DY experiment. The blades were sampled on 50 (main stem), 64 (main stem) and 84 (tillers) DAS to obtain a wide range of ranks and SPAD values. The dried blades were ground using pestle and mortar and sieved in a 1 mm sieve, removing the veins from the sample. The resulting powder of leaf mesophyll was weighed per leaf. Multiple leaf samples were combined in SPAD intervals to obtain sufficient material for analysis. For the lowest [5; 10 [interval, it was necessary to combine blades from plants grown under the 6h-6h and 14h-10h light-dark cycles to obtain sufficient material for analysis.

Per SPAD interval, approximately 200 mg of sample was weighed analytically and total N concentration (%) was analyzed using a SNC analyzer (Primacs SNC 100-IC-E, Skalar Analytical BV, Breda, The Netherlands), with combustion of the sample at 1100°C. Of the same samples, approximately 1 g was weighed analytically in 100 ml demineralized water. The mixture was shaken at 100 rpm for 30 minutes. The NO_3_^-^ concentration (mg kg^-1^) was measured after a 10-fold dilution using an ICS-1100 (Dionex, Thermo Scientific, USA), with a AS22 column (Dionex Ionpac, Thermo Scientific, USA) using a 4.5 mM Na_2_CO_3_/1.4 mM NaHCO_3_ mobile phase.

In the HL + C_e_ + DY experiment, a total of 36 leaves of the 6h-6h light-dark cycle were ground for analysis. For 30 leaves from the main stem, where leaf area could be estimated, the N and NO_3_^-^ concentrations corresponding to each SPAD interval were combined with the leaf area and the weight of the resulting powder to calculate N and NO_3_^-^ content per unit of leaf area (mg m^-^²).

### 2.5 Statistical analysis

Statistical analysis was performed in RStudio (Posit team, 2023) using R version 4.3.0 (R Core Team, 2023). The ‘ggplot2’ package (Wickham, 2016) was used for visualization. To test for significant differences between leaf groups, and to fit the relationship between SPAD and dry weight per leaf area on the one hand and nitrogen and (nitrogen – nitrate) content on the other hand, linear mixed models, including a random effect for plant id, were fitted using the ‘lmerTest’ package (Kuznetsova et al., 2017). Model diagnostics are shown in **Supplementary Table S3**. Linear model assumptions were tested visually and numerically (Peña and Slate, 2006). Of the relationships, assumptions were only met for the one between leaf SPAD value and N – NO_3_^-^ content. Linear models, power models, and ln transformed models were fitted. Based on significance of the coefficients, regression coefficients and visual inspection of the correct functional form, a linear relationship was selected. If the assumptions for linear regression were not met, Pearson’s correlation was shown using the ‘smplot2’ (Min and Zhou, 2021) package.

Error propagation was applied to the calculations of N and NO_3_^−^ content per unit leaf area, based on measurement uncertainties in leaf length, width, dry weight, and the chemical analyses of N and NO_3_^−^.

## 3 Results

**Figure 3:**
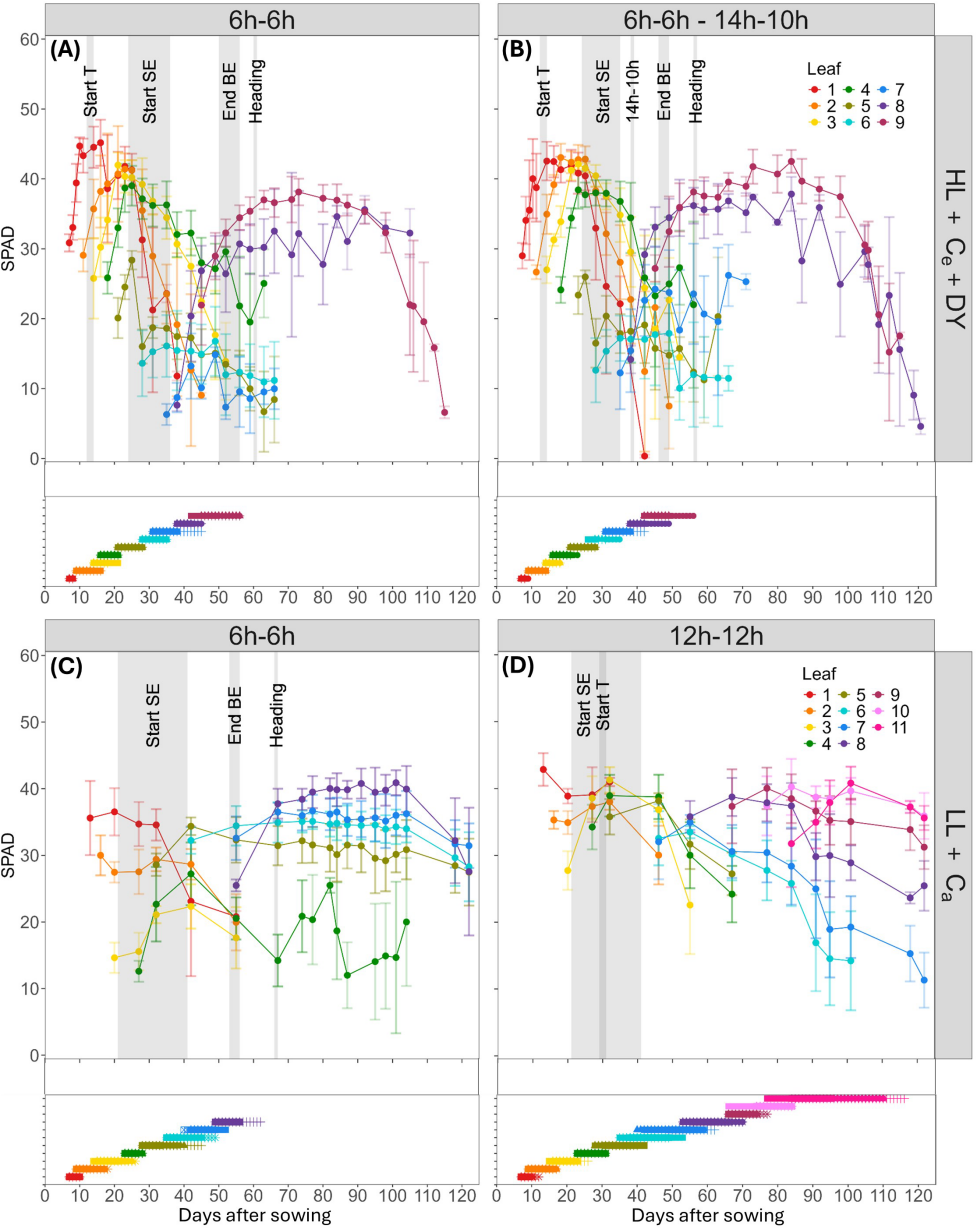
Dynamics of mean SPAD values (top graph in each panel) and elongation duration (bottom graph in each panel) of wheat blades on the main stem in relation to phenological events and during pre-anthesis development. SPAD evolution and blade elongation at high light intensity and elevated CO_2_ conditions, with dynamic light fixtures (HL + C_e_ + DY) (n=4), with **(A)** the 6h-6h light-dark cycle, and **(B)** the 6h-6h followed by a 14h-10h light-dark cycle, and low light intensity and ambient CO_2_ (LL + C_a_) conditions (n=6) with **(C)** the 6h-6h light-dark cycle, and **(D)** the 12h-12h light-dark cycle. Bars indicate standard deviation. Measurements were performed on distinct samples (biological replicates). A grey background color indicates the interval in which a phenological event took place: start of tillering (Start T), start of stem elongation (Start SE), end of blade elongation (End BE) and heading. The transitioning day for the change in light-dark cycle from 6h-6h to 14h-10h in the HL + C_e_ + DY experiment is indicated by 14h-10h. Flag leaves are leaf 11 for the 12h-12h light-dark cycle, and leaf 8 for the 6h-6h light-dark cycle of the LL + C_a_ experiment, and leaf 9 for the HL + C_e_ + DY experiment.

To investigate the effect of a 6h-6h light-dark cycle on spring wheat development and leaf greenness, regular SPAD measurements were related to key developmental phases, especially the onset of stem elongation. In the HL + C_e_ + DY experiment, the typical pattern of increasing leaf SPAD values up to a peak, followed by a subsequent decrease was observed (**Figures 3A, B**). In the 6h-6h cycle, maximum main stem SPAD values ranged from 34 to 46. However, around the onset of stem elongation, significantly lower maximum SPAD values were observed for leaves five to seven on the main stem (p < 0.05), with values of 19.1 ± 3.0 and 15.9 ± 2.1. Between 21 to 25 DAS, SPAD values for leaf five increased to 28.4 ± 1.3, but after 28 DAS, again around the start of stem elongation, these values dropped below 20 (**Figure 3A**). In the 14h-10h treatment, similar values and dynamics were observed, again maximum SPAD values of leaves five to seven were significantly lower (p < 0.05). Leaves one to four exhibited maximum SPAD values between 39 and 43, while leaf five, despite its initial increase, and leaf six showed contrastingly lower values around 20. Leaf seven, developing when the transition from the 6h-6h to the 14h-10h cycle was carried out, revealed a higher maximum SPAD value of 26.5 ± 7.3, compared to the 6h-6h cycle (**Figure 3B**).

Similarly, under a lower light intensity and CO_2_ concentration in a 6h-6h cycle, significantly lower SPAD values were observed in the second to fourth leaf (p < 0.05), which appeared around the start of stem elongation, with maximum values between 20 and 30. On the other hand, maximum SPAD values, ranging between 34 and 43, were similar in the 6h-6h and the 12h-12h cycles. In the LL + C_a_ treatment, the typical pattern of increasing SPAD values up to a peak, followed by a subsequent decrease, was not consistently observed. For emerging blades, measurements started too late to observe the increase in SPAD. For senescing leaves in this 6h-6h cycle, the decrease was observed for leaves one to three, around 40 DAS, while for leaves five to eight, a slight decrease was observed starting around 110 DAS (**Figure 3C**). In the 12h-12h cycle, a clear decrease was observed up to eight leaves, while the younger leaves started to decrease slightly around 115 DAS (**Figure 3D**). Whether this decrease persisted could not be observed, because measurements stopped at 122 DAS.

The flag leaf maximum SPAD values were comparable across both experiments, with values of 38 and 40 for the 6h-6h and 14h-10h cycles in the HL + C_e_ + DY experiment (leaf 9), and 42 and 40 for the 6h-6h (leaf 8) and 12h-12h cycles (leaf 11) in the LL + C_a_ experiment. Both experiments showed that leaf greenness in all shortened 6h-6h light-dark cycles, with average maximum SPAD readings of 37.7 ± 3.1 (n = 28; LL + C_a_), 40.4 ± 4.7 (n = 24; HL + C_e_ + DY – 6h-6h) and 41.3 ± 2.7 (n = 24; HL + C_e_ + DY – 14h-10h), was comparable to that in the 12h-12h cycle, with an average value of 38.3 ± 5.6 (n=68). However, leaves that developed around the stem elongation phase showed substantially lower maximum values, with averages of 26.9 ± 3.9 (n = 18; leaves 2 – 4; LL + C_a_), 21.1 ± 5.9 (n = 12; leaves 5 – 7; HL + C_e_ + DY – 6h-6h), and 24.1 ± 5.4 (n = 12; leaves 5 – 7; HL + C_e_ + DY – 14h-10h).

**Figure 4:**
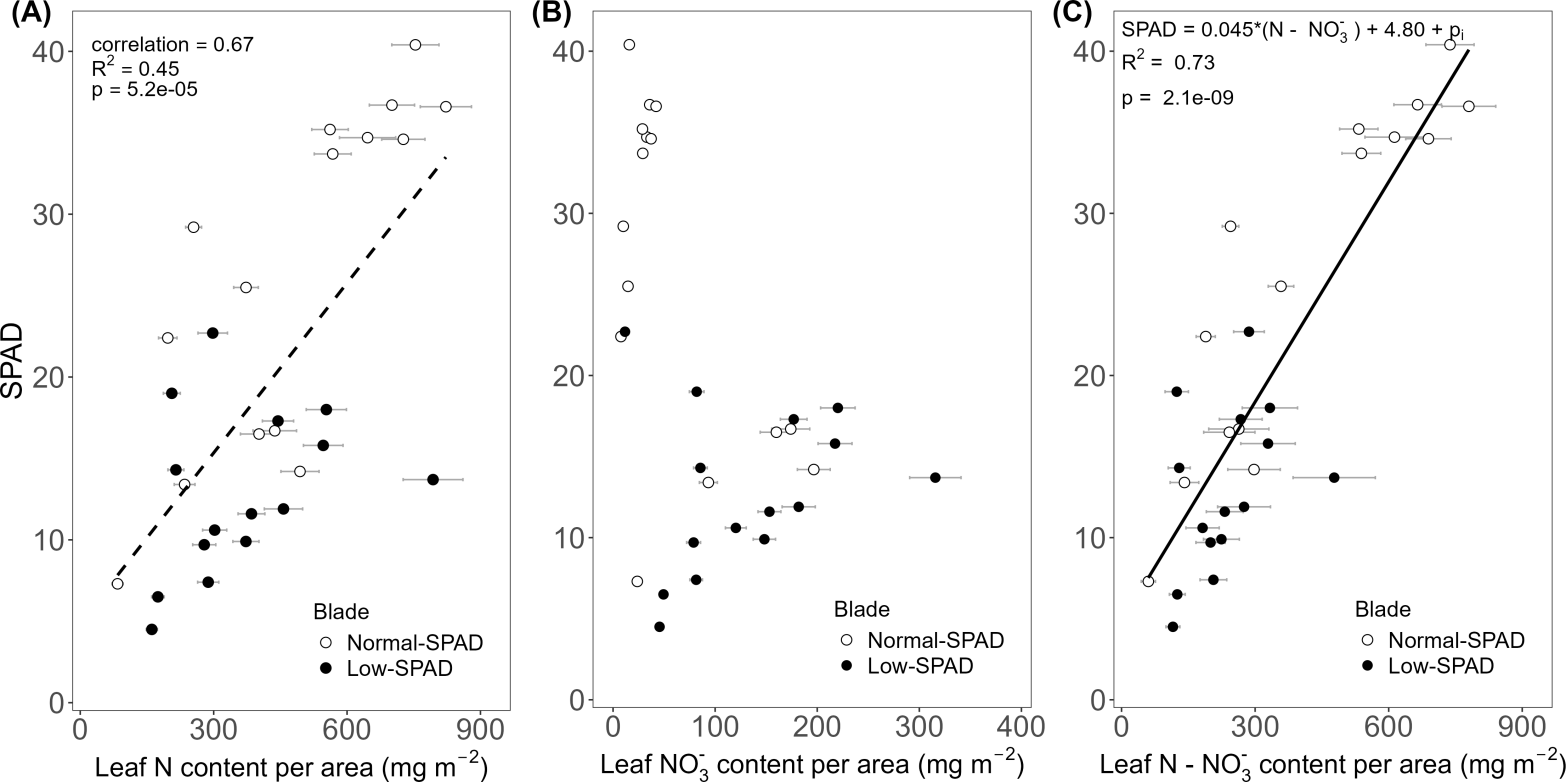
Relationship between leaf SPAD and leaf nitrogen and nitrate content per unit leaf area. SPAD values measured on main stem blades plotted against **(A)** estimated total nitrogen (N) content, **(B)** estimated nitrate (NO_3_^-^) content and **(C)** estimated nitrogen minus nitrate (N - NO_3_^-^) content per unit leaf area. Based on data from the high light intensity and elevated CO_2_ experiment, with dynamic light fixtures (HL + C_e_ + DY) with the 6h-6h light-dark cycle. Each circle corresponds to a blade harvested during destructive measurements. Open circles show normal-SPAD leaves (numbers 1 – 4, 8 and 9), closed circles show low-SPAD leaves (numbers 5 – 7). Linear correlations are shown by a dashed line, linear regressions by a full line. Error bars reflect error propagation based on measurement uncertainties. If no error bar is visible, it falls within the bounds of the circle representing the data point.

To relate the contrasting SPAD dynamics observed in wheat leaf blades grown under the 6h-6h light-dark cycle in the HL + C_e_ + DY experiment to leaf N status, blade N content per unit leaf area was compared to the corresponding SPAD values. This was done for leaves three to nine on the main stem of the destructively sampled plants on 50 and 64 DAS. In these data, two groups emerged. The first group of leaves showed lower N contents, roughly between 200 and 600 mg m^-2^, with SPAD values ranging from 5 to 20, while the second group showed higher N contents, reaching up to 800 mg m^-2^, with SPAD values ranging from 20 to 40 (**Figure 4A**). These two groups corresponded to leaves five to seven, the low-SPAD leaves (closed circles), and leaves three, four, eight and nine, the normal-SPAD leaves (open circles), respectively. A positive linear correlation was observed between N content and SPAD values (R² = 0.45, p < 0.05, correlation coefficient = 0.67). To further explore this correlation, the relationship with leaf NO_3_^-^ content was analyzed (**Figure 4B**). Again, two groups were observed: one with SPAD values above 20 and low nitrate values below 50 mg m^-2^, corresponding to leaves three, four, eight and nine (open circles), and the second one with SPAD values below 20 and high nitrate levels above 50 mg m^-2^, corresponding to leaves five to seven (closed circles). By subtracting NO_3_^-^ from the total N content, the resulting significant linear relationship (p < 0.05) with SPAD yielded a higher R² value of 0.73 (**Figure 4C**).

**Figure 5:**
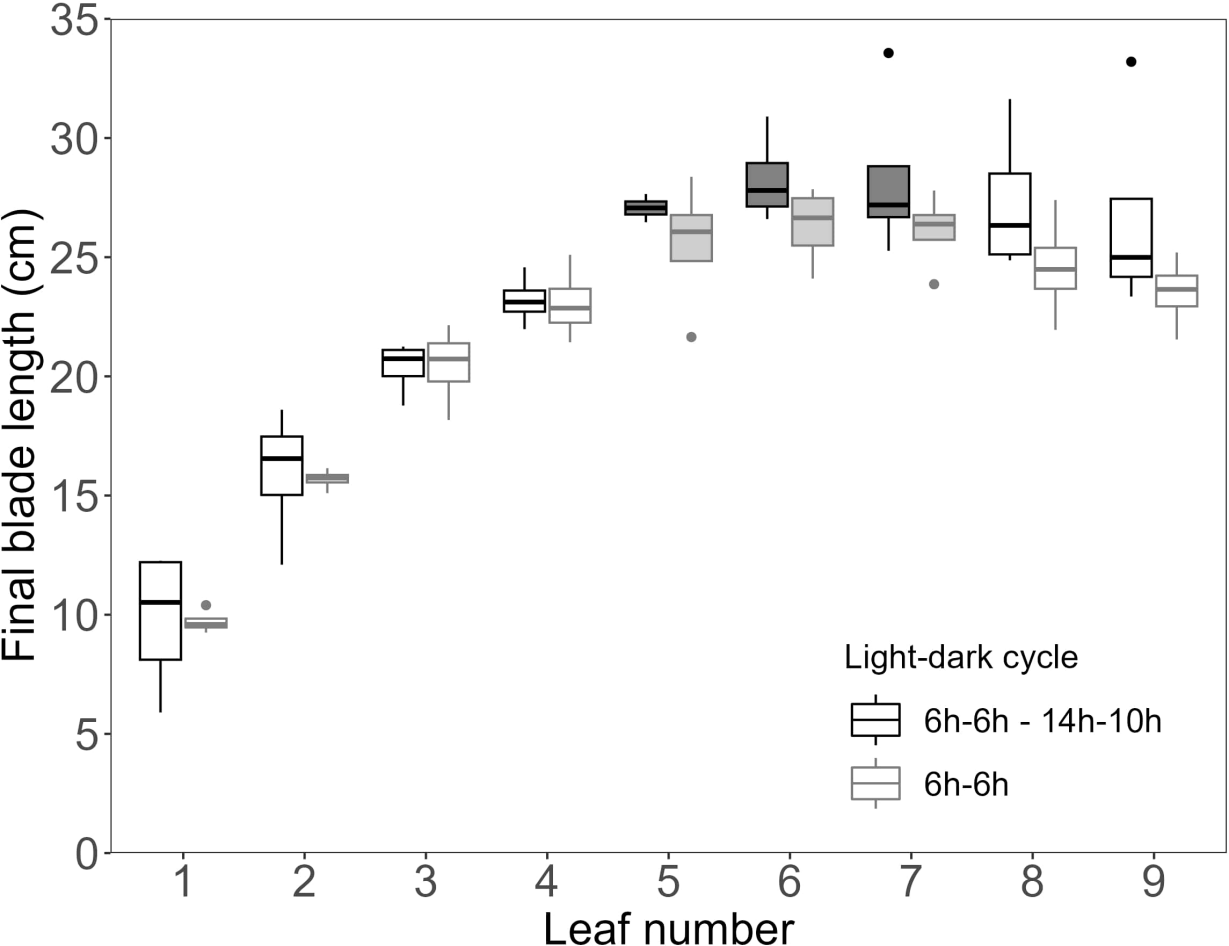
Final length of wheat main stem blades for the high light intensity and elevated CO_2_ experiment, with dynamic light fixtures (HL + C_e_ + DY). Light grey boxplots indicate the 6h-6h light-dark cycle (n=4), and dark grey boxplots indicate the 6h-6h followed by the 14h-10h light-dark cycle (n=4). Measurements were performed on distinct samples (biological replicates). Filled boxplots indicate nitrate accumulating leaves.

To test if also blade length was impacted by the 6h-6h light-dark cycle, final blade length was plotted against leaf number (**Figure 5**). It was observed that a reduced leaf greenness did not result in a reduced final leaf length. Specifically, the NO_3_^-^ accumulating leaves, with a low SPAD value, showed the highest final blade length.

**Figure 6:**
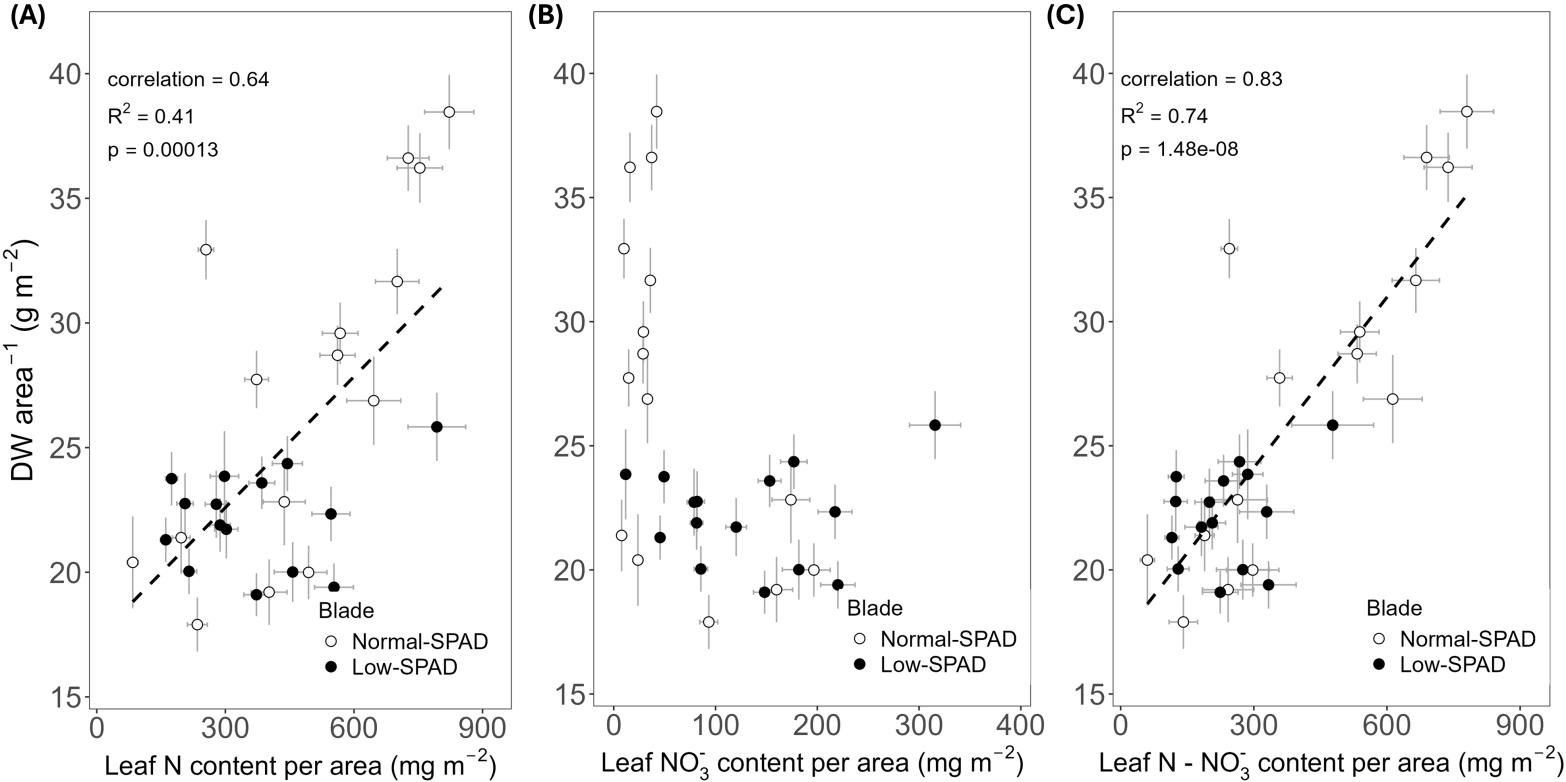
Dry weight per leaf area plotted against **(A)** estimated total nitrogen (N), **(B)** estimated nitrate (NO_3_^-^) and **(C)** estimated nitrogen minus nitrate (N - NO_3_^-^) content per unit leaf area, for the high light, elevated CO_2_ and dynamic light fixtures experiment (HL + C_e_ + DY) with the 6h-6h light-dark cycle. Each symbol corresponds to a blade harvested during destructive measurements. Open circles show normal-SPAD leaves. Closed circles show low-SPAD leaves. Dashed lines indicate linear correlations. Error bars reflect error propagation based on measurement uncertainties. If no error bar is visible, it falls within the bounds of the circle representing the data point.

**Figure 7:**
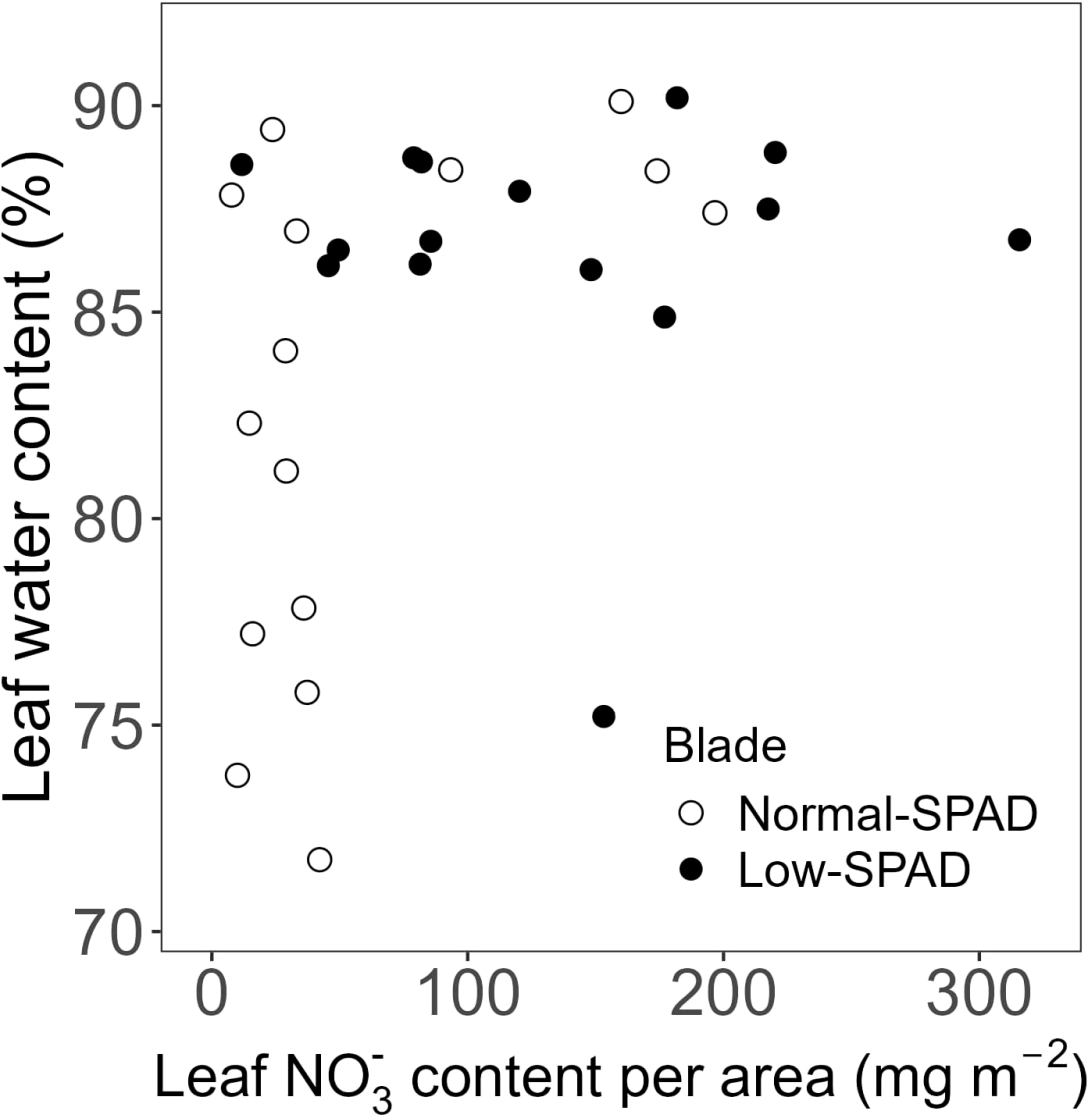
Leaf water content plotted against estimated nitrate (NO_3_^-^) content per unit leaf area, for the high light, elevated CO_2_ and dynamic light fixtures experiment (HL + C_e_ + DY) with the 6h-6h light-dark cycle. Each symbol corresponds to a blade harvested during destructive measurements. Open circles show normal-SPAD leaves. Closed circles show low-SPAD leaves.

As reduced leaf greenness did not affect the length of leaves five to seven, we assessed whether it impacted leaf DW per unit of leaf area (**Figure 6**). While the DW of leaves five to seven, the low-SPAD leaves, varied roughly between 20 and 25 g m^-2^ across N contents from 200 to 600 mg m^-2^, the other leaves showed a pattern where higher DW corresponded with higher N contents. A significant positive correlation between DW area^-1^ and N content per unit leaf area was observed (p < 0.05; R² = 0.41; correlation coefficient = 0.64; **Figure 6A**). The NO_3_^-^ content data clearly distinguished the two groups of leaves (**Figure 6B**). Leaves five to seven had a DW below 25 g m^-2^, with NO_3_^-^ contents reaching up to about 220 mg m^-2^, while the other leaves generally had NO_3_^-^ contents below 100 mg m^-2^ and DW area^-1^ reaching up to 40 g m^-2^. The difference between the two groups was also clear from the increased water content of the NO_3_^-^ accumulating leaves (**Figure 7**). When subtracting the NO_3_^-^ from the total N content, a more clear significant linear correlation between blade dry weight and N – NO_3_^-^ content was observed (p < 0.05; R² = 0.74; correlation coefficient = 0.83; **Figure 6C**).

**Figure 8:**
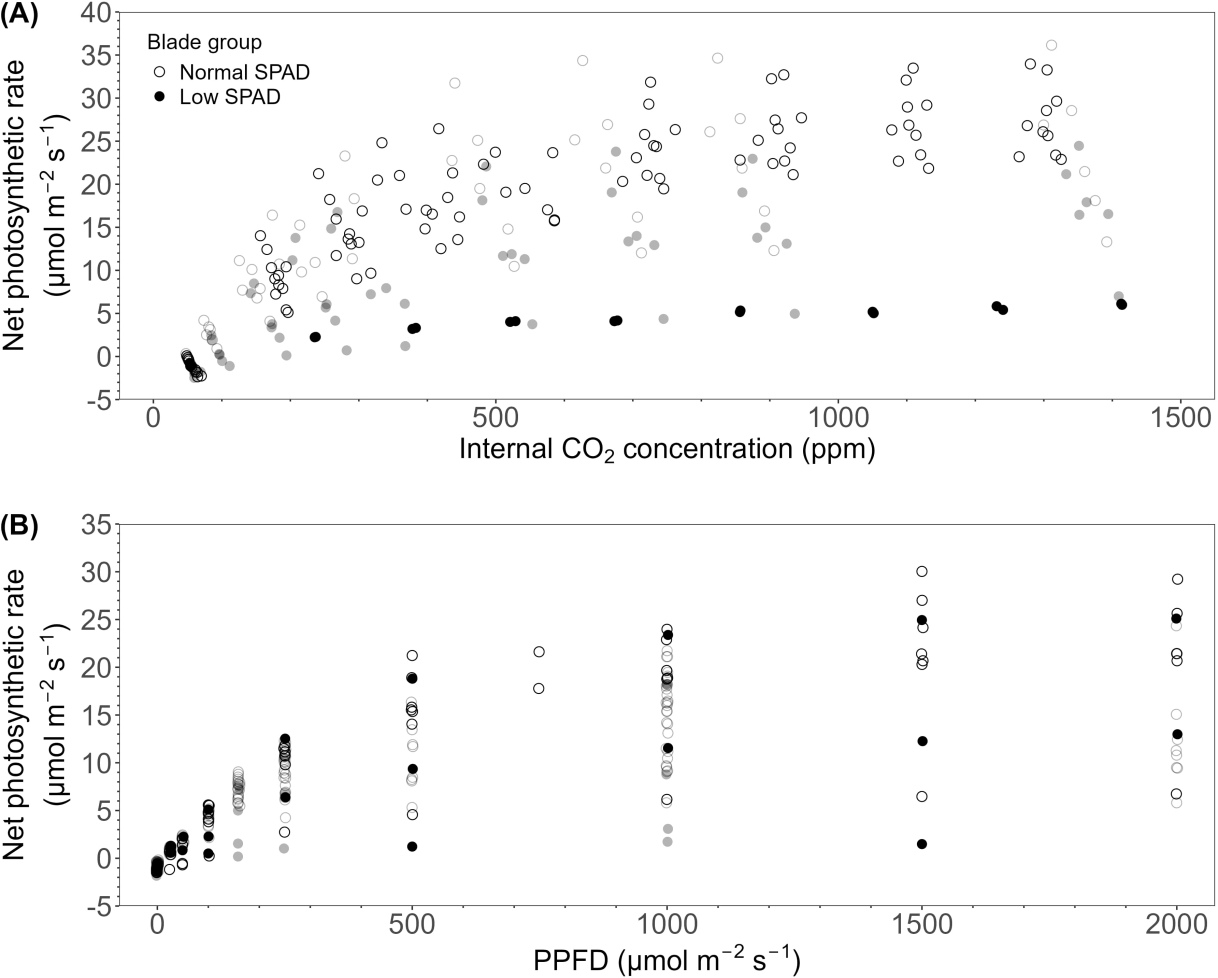
CO_2_ response curves (AC_i_) and light response curves (LRC) for wheat blades sampled in the high light, elevated CO_2_ concentration with dynamic light fixtures experiment (HL + C_e_ + DY; black circles) and the low light and ambient CO_2_ concentration experiment (LL + C_a_; grey circles). **(A)** CO_2_ response curves showing blade net photosynthetic rate plotted against leaf internal CO_2_ concentration. **(B)** Light response curves showing blade net photosynthetic rate plotted against photosynthetic photon flux density (PPFD). Open circles indicate blades with a normal SPAD value (low nitrate content), closed circles indicate blades with a low-SPAD value (high nitrate content). Only blades grown under a 6h-6h light-dark cycle are displayed.

The effect of reduced leaf greenness on source capacity was assessed through photosynthesis measurements. In the HL + C_e_ + DY experiment (black circles), two groups of AC_i_ curves were observed (**Figure 8A**). The maximum photosynthetic rate of leaves five to seven (low-SPAD leaves), laid around 5 µmol m^-2^ s^-1^, compared to values between 22 and 34 µmol m^-2^ s^-1^ for the normal-SPAD leaves of comparable leaf ages (**Supplementary Figure S4**). In the LL + C_a_ experiment (grey circles), maximum photosynthetic rates between 5 and 15 µmol m^-2^ s^-1^ were observed for leaves two to four, the low-SPAD leaves. Two of the curves measured on leaf 4 showed higher photosynthetic rates, roughly around 20 µmol m^-2^ s^-1^, and corresponded to higher SPAD values of 28.2 and 28.6, compared to values between 17 and 25 for the other leaves. However, when analyzed by leaf age, these curves fall below those of normal-SPAD leaves of comparable age (**Supplementary Figure S5**). For leaves with normal-SPAD values, rates between 13 and 36 µmol m^-2^ s^-1^ were observed. Looking at LRC curves, such a distinction was not clearly observed for either experiment (**Figure 8B**).

## 4 Discussion

Growing spring wheat under a 6h-6h light-dark cycle accelerated heading and enabled satisfactory yield (**Supplementary Figure S2**; Clauw et al., 2024). Nevertheless, leaf physiology around the onset of stem elongation was impacted, as demonstrated by the SPAD measurements taken on main stem leaves (**Figure 3**). Under a light intensity of about 670 µmol m^-2^ s^-1^ and an elevated CO_2_ concentration of 696 µmol m^-2^ s^-1^ (HL + C_e_ + DY), the 6h-6h light-dark cycle resulted in a pronounced dip in SPAD values. Specifically, leaves five, six and seven showed maximum values below 20, compared to maximum values around 40 for the other leaves. This pattern was also observed in the 6h-6h to 14h-10h treatment, with the exception of leaf seven, which showed increasing SPAD values after the switch to a 14h daylength. Under a lower light intensity of about 93 µmol m^-2^ s^-1^ and an ambient CO_2_ concentration of 479 ppm (LL + C_a_), this dip was also observed, however, less pronounced, with leaves two, three and four having SPAD values below 30. Under identical environmental conditions, except for a longer light-dark cycle of 12h-12h, all measured SPAD values were above 30.

### 4.1 Stem elongation affects SPAD values and leaf N status

Positive relationships between SPAD and N concentration or content per unit of leaf area have been reported for multiple species in the literature (Jinwen et al., 2011; Xiong et al., 2015; Mehrabi and Sepaskhah, 2022). In this work, a significant positive linear correlation between SPAD values and leaf N content per area was observed (**Figure 4A**). However, when first examining raw total N concentrations, (on a mass basis, %) across SPAD intervals, the highest concentration was observed in the low SPAD interval of 10 to 20 (Clauw et al., 2025), sampled mainly from leaves five to seven, which are in this study referred to as the low-SPAD leaves. When specifically analyzing NO_3_^-^ concentrations of the same samples, the leaves with SPAD readings below 20 showed a strikingly higher NO_3_^-^ content, calculated by converting the raw NO_3_^-^ concentration to NO_3_^-^ content (mg per unit of leaf area). This reached up to about 200 mg m^-2^, compared to about 50 mg m^-2^ for the other leaves (**Figure 4B**). As NO_3_^-^ is included in the Dumas method for N analysis, this explained the observed higher raw N concentration values. By calculating the organic N content, subtracting the NO_3_^-^ content from the total N content, the assumptions for fitting a linear regression, rather than a correlation, between SPAD and organic N content were met (**Figure 4C**). As chlorophyll content is related to organic N availability, the lower organic N contents, coupled to nitrate accumulation, explain the low SPAD readings on leaves five to seven.

The positive linear regression indicated that low-SPAD leaves had a low organic N content, likely due to NO_3_^-^ accumulation. As a result, the vertical pattern of leaf organic N content deviated from the generally observed pattern that follows the gradient of absorbed light, where lower canopy leaves have a lower organic N content (Dreccer et al., 2000; Kichey et al., 2006). In the HL + C_e_ + DY experiment, at 42 DAS for example, SPAD values from the topmost to the bottommost leaf were 20.4 (leaf 8), 13.2 (leaf 7), 15.3 (leaf 6), 17.3 (leaf 5), 32.3 (leaf 4), 27.5 (leaf 3) and 12.7 (leaf 2). On that day, leaf 4, positioned relatively low in the canopy, had the highest SPAD value. This observation is not likely the result of N redistribution to upper leaves, but rather reflects the compromised N status of leaves five to seven (the low-SPAD leaves), which never reached maximum SPAD values comparable to those of the other leaves.

During reproductive development, source-sink relationships change and mature leaves, initially sucrose sources for developing leaves, now become sources for the developing apex and elongating stem (Gol et al., 2017), an important sink for carbohydrates for later use in grain filling (Blum, 1998). Patrick (1972) investigated the distribution of assimilates during stem elongation in wheat and found that from a source leaf, assimilation transport was greatest to the internodes immediately below and above the leaf. This is in accordance with our observations: destructive measurements showed that significant elongation started at the fifth internode, above the first low-SPAD leaf. Similarly, in the LL + C_a_ experiment, elongation was first observed at the fifth internode. In this case, however, the second, third and fourth leaves showed unexpectedly lower SPAD values. The differing environmental conditions between the two experiments may have influenced the dynamics. Because of the static light fixtures, older leaves intercept lower light intensities compared to younger leaves, growing closer to the lights (**Supplementary Figure S3**). The low SPAD values of leaves two and three, developing before the start of significant internode elongation, may thus be the result of lower light intensities intercepted by these leaves, possibly combined with the switch to a generative apex. Leaves two and three, at a respective distance of about 70 and 64 cm from the light, received light intensities of around 200 µmol m^-2^ s^-1^. Although leaf five was initially located at an estimated distance of 54 cm from the light and did not intercept a higher light intensity, its exposure likely increased once stem elongation began. This is supported by the observed exponential rise in light intensities at distances below 50 cm. The resulting increase in light availability may have supported the physiological activity of leaf five, as reflected in its higher SPAD values.

Interestingly SPAD values of the flag and penultimate leaf, located near the peduncle and second internode, increased compared to the low-SPAD leaves. This is in accordance with observations by Ehdaie et al. (2006), who reported that, in contrast to the peduncle and second internode, the lower internodes have a higher specific weight, and averaged across cultivars and treatments, more than 50% of the stem’s dry matter can be stored in the lower internodes, typically comprising two to four internodes, depending on the cultivar. In a field study, Grover et al. (1978) observed higher nitrate reductase activity in the flag leaf blade compared to the penultimate and the third highest leaf blade, along with lower moisture percentages and NO_3_^-^ contents in the flag leaf relative to the third blade. One of their explanations is the differential impact of the developing sink on the leaf blades, which provides a plausible explanation for our findings. After anthesis, when the final number of grains, an important factor for final yield, has already been determined during stem elongation, the source strength shifts to support grain filling, further building towards final yield (Murchie et al., 2023). As this process relies primarily on the flag and penultimate leaves, the higher SPAD values and photosynthetic rates of these leaves observed under both light-dark cycles (**Figure 8**) may have supported productivity. This is reflected in comparable dry weights per grain under both the 6h-6h and 14h-10h cycles (Clauw et al., 2024).

### 4.2 Short light-dark cycle affects SPAD values and leaf N status

The most pronounced deviation from the typical leaf greenness pattern was observed in the 6h-6h light-dark cycles (**Figures 3A-C**). Apart from slightly lower values in leaves six and seven, this deviation was not present in the 12h-12h light-dark cycle (**Figure 3D**). Under short light periods, Matt et al. (1998) observed simultaneous carbon and nitrogen deficiencies in tobacco plants. Under a 6h-18h light-dark cycle, the plants showed very high nitrate levels, reduced protein and chlorophyll levels, and very low sugar levels. The authors suggested that one reason for this observation is the stimulation of starch synthesis during short days, which reduces the carbon available for nitrate reduction. A shorter photoperiod indeed leads to higher rates of starch synthesis and, conversely, lower rates of sucrose synthesis (Smith and Stitt, 2007). This may offer a plausible explanation for the current experiment, as the difficulties in N status emerged around stem elongation, a developmental phase characterized by high carbohydrate demand. Moreover, under a short night, Arabidopsis has been shown to degrade starch too slowly to fully deplete its reserves, leaving up to 40% of the starch unused by the end of the night (Graf et al., 2010). However, this hypothesis remains speculative. To validate this hypothesis, future experiments should include measurements of starch and carbohydrate dynamics, together with measurements of nitrate reductase activity.

Nevertheless, it has been shown that a mismatch between the light-dark cycle and the plant’s free-running period leads to decreased chlorophyll contents and lower rates of CO_2_ assimilation (Dodd et al., 2005). In a number of horticultural crops, SPAD values have been shown to decrease under multiple light-dark cycles in a 24 hour time period compared to one light-dark cycle. In lettuce, higher SPAD values, and photosynthetic rates have been reported in a 12h-12h light-dark cycle compared to multiple 6h-6h and 3h-3h light-dark cycles (Zhou et al., 2020). Similarly, lower chlorophyll a + b concentrations have been observed in tomato and cucumber seedlings subjected to two or three light-dark cycles per day compared to a single cycle (García-Caparrós et al., 2020). In addition, in tomato, a higher number of light-dark cycles compared to one 24h-cycle led to reduced chlorophyll and carotenoid concentrations, lower photosynthetic rates, and decreased starch and soluble sugar contents (Yuan et al., 2025). In our experiments, AC_i_ curves reflected the differences between the low- and normal-SPAD leaves, showing overall lower values for the low-SPAD leaves in the HL + C_e_ + DY and LL + C_a_ experiments, which is in line with the literature (**Figure 8**). In contrast, the normal-SPAD leaves, also growing under multiple light-dark cycles per day, showed higher photosynthetic rates coupled to the higher SPAD values. In this study, the development of an influential sink negatively affected leaves growing at the same time, resulting in clear difficulties only in these leaves. The studies cited above investigated lettuce and young plants, which were negatively affected by the multiple light-dark cycles, even in the absence of a generative sink. Potentially, the influence of multiple light-dark cycles might increase when these crops are grown until a generative stage, suggesting the potential of experiments performed until the generative stages. Differences between normal- and low-SPAD leaves were less distinct in LRCs of the HL + C_e_ + DY experiment (**Figure 8B**). This can be explained by the SPAD value of the LRC curve with the highest values, which was 36.6, compared to 17.8 and 5.2 for the other two low-SPAD leaves. In the LL + C_a_ data also two LRCs can be distinguished with higher values compared to the other low-SPAD leaves (**Figure 8B**). To further confirm this, a higher number of measurements should be carried out.

Following the switch to the 14h-10h light-dark cycle, SPAD values in leaf seven increased. Graf et al. (2010) observed that by extending the night in the 8.5h-8.5h cycle until 26 hours after dawn, starch degradation continued, with reserves fully depleted 23 hours after dawn. Further, Matt et al. (1998) observed that nitrate reductase activity increased when short-day-grown plants were transferred to long days. Changing to the plants’ free running photoperiod likely helped to rebalance carbohydrate levels and starch allocation, thereby supporting normal N metabolism during stem elongation. This phase is critical for flower development, and consequently for determining the final grain number, and relies on maximum radiation interception and photosynthesis (Murchie et al., 2023). The lower photosynthetic rates observed in the low-SPAD leaves (**Figure 8**) around stem elongation, may therefore have negatively impacted productivity. In fact, switching from a 6h-6h light-dark cycle to a 14h-10h cycle increased the number of grains on the main stem (Clauw et al., 2024). The improved N metabolism and associated productivity gains demonstrate the potential of aligning photoperiod regimes to the plant’s developmental stages when implementing short light-dark cycles.

Accumulation of NO_3_^-^ in plant tissues under photosynthesis-limiting conditions is a well know mechanism to compensate for the reduced levels of sugars and organic acids (Blom-Zandstra and Lampe, 1985; Lieffering et al., 1996; Andrews, 2005). In dark-grown wheat seedlings, NO_3_^-^ concentrations, calculated from the molar concentrations, ranging between 24800 mg kg^-1^ to 37200 mg kg^-1^ have been reported (Lieffering et al., 1996). These values are comparable to those observed in our low-SPAD leaves (19300 mg kg^-1^; Clauw et al. (2025)). Because NO_3_^-^ may function as an osmolyte (Blom-Zandstra and Lampe, 1985; Andrews, 2005), its accumulation may have increased turgor pressure in the leaves, thereby driving turgor-driven growth and promoting leaf elongation (Lockhart, 1965; Steppe et al., 2006; Coussement et al., 2021). Consequently, in our experiment, the low-SPAD leaves showed both higher water content (**Figure 7**) and higher final leaf blade length (**Figure 5**), possibly due to NO_3_^-^ accumulation. The resulting pattern of final blade length coincides with what has been typically observed for gramineous species (Evers et al., 2005). The low-SPAD leaves showed a lower dry weight per unit of area (**Figure 6**), implying lower tissue density and possibly reduced mechanical strength.

Although environmental conditions, specifically CO_2_ concentration and lamp setup, differed between the two experiments, the consistent observation of reduced SPAD values in leaves developing around the start of stem elongation under a 6h-6h light-dark cycle underscores the generality of this novel phenomenon. Moreover, the varying extent of the decline suggests that its expression may be modulated by the specific background conditions. Increased CO_2_ has been shown to reduce leaf N concentration and leaf chlorophyll content in wheat (Wang et al., 2013). The decline in SPAD readings was indeed more pronounced in the HL + C_e_ + DY experiment compared to the LL + C_a_ experiment, probably due to the elevated CO_2_ concentrations. However, overall SPAD values in this treatment were not lower than those observed under ambient CO_2_ concentrations. Under the static lamp setup in the LL + C_a_ experiment, higher-ranking leaves received more light due to their closer proximity to the lamps. This may explain the slightly higher maximum SPAD values measured in leaves developed after, compared to before, the start of stem elongation. Nevertheless, leaves two, three and four, developing around the start of stem elongation, still showed a decline in SPAD values, albeit to a lesser extent than in the HL + C_e_ + DY experiment. The observation of a SPAD decline in both experiments, despite their differing environmental conditions, reinforces the physiological relevance of the response to a 6h-6h light-dark cycle.

### 4.3 Conclusion

Growing wheat under a 6h-6h light-dark cycle, offering potential energy savings in controlled environments, has been shown to accelerate heading, thereby increasing the number of growth cycles possible in one year and enhancing profitability.

The observed growth and development indicate that spring wheat can grow normally under a 6h-6h light-dark cycle. However, during the stem elongation phase, this short photoperiod caused imbalances in source-sink relationships, which negatively affected leaf N status. Significantly lower SPAD readings revealed nitrate accumulation and reduced photosynthetic rates. It is striking that, despite the broad effects of the light-dark cycle on physiology and development, the most pronounced disruptions were confined to the stem elongation phase. Transitioning to a longer light-dark cycle at this phase improved SPAD values and photosynthetic rates. These findings suggest that tailoring the light-dark cycle to the plant’s developmental stage shows potential for supporting both productivity and quality, while maintaining energy efficiency.

## Data Availability

The datasets presented in this study can be found in online repositories. The names of the repository/repositories and accession number(s) can be found below: http://doi.org/10.5281/zenodo.14529253.
