# Supplementary material for: A 6-hour light-dark cycle reduces photosynthesis and leaf greenness in spring wheat at stem elongation through nitrate accumulation
Source: Front Plant Sci. 2025 Sep 12;16:1655271. doi: 10.3389/fpls.2025.1655271 (PMC12463616; doi:10.3389/fpls.2025.1655271)
Supplement: Supplementary file 1 [file DataSheet1.docx]

Supplementary Material

**Supplementary Table S1.** Composition of the stock solution used for the nutrient solution in the two experiments.

| LL + C_a_ and HL + C_e_ + DY | | |
| --- | --- | --- |
| Concentration (g L^-1^) | | |
| Ca(NO_3_)2.4H_2_O | 127.5 |  |
| KNO_3_ | 40.5 |  |
| KH_2_PO_4_ | 20.5 |  |
| MgSO_4_.7H_2_O | 59 |  |
| K_2_SO_4_ | 35 |  |
| MnSO_4_.H_2_O | 0.22625 |  |
| Na_2_MoO_4_.2H_2_O | 0.015 |  |
| NH_4_NO_3_ | 9.5 |  |
| H3BO_4_ | 0.3575 |  |
| ZnSO_4_.7H_2_O | 0.0275 |  |
| CuSO_4_.5H_2_O | 0.006375 |  |

**Supplementary Table S2.** Average measured electrical conductivity (EC, µS cm^-1^), pH and dissolved oxygen (DO, mg L^-1^) of the nutrient solution on the cultivation tray, measured before and after adding new solution to the table. Within light-dark treatments, statistical differences between mean values of these parameters, before and after adding fresh nutrient solution were tested. Statistical differences between the mean values of EC, pH and DO were tested using a student’s t-test. Different letters indicate significant differences (p < 0.05).

|  | 6h-6h | | 14h-10h | |
| --- | --- | --- | --- | --- |
|  | Before | After | Before | After |
| EC (µS cm^-1^) | 2027 ± 336^a^ | 1925 ± 235^a^ | 2117 ± 370^a^ | 1952 ± 224^a^ |
| pH | 6.7 ± 0.3^a^ | 6.6 ± 0.4^a^ | 6.7 ± 0.1^a^ | 6.6 ± 0.3^b^ |
| DO (mg L^-1^) | 1.46 ± 0.55^a^ | 4.05 ± 0.92^b^ | 1.18 ± 0.19^a^ | 3.67 ± 0.85^b^ |

**Supplementary Table S3.** Model diagnostics of the used linear mixed models.

| Maximum SPAD | R equation: SPAD_max_ ~ LeafGroup + (1 \| PlantID) | | | |
| --- | --- | --- | --- | --- |
| Experiment - treatment | Fixed effects - estimations | Fixed effects – 95% CI | Random effects | R² |
| HL + C_e_ + DY - 6h-6h  (n = 36) | Intercept: 40.4 (p < 2e-16)  Slope: -19.29 (p = 2.2e-12) | [38.36; 42.44]  [-22.83; -15.75] | Variance = 0.00  Std. Dev. = 0.00 | Marginal: 0.76  Conditional: 0.76 |
| HL + C_e_ + DY - 14h-10h  (n = 36) | Intercept: 41.34 (p = 1.92e-09)  Slope: -17.25 (p = 4.88e-14) | [39.83; 42.85]  [-19.86; -14.63] | Variance = 0.16  Std. Dev. = 0.40 | Marginal: 0.83  Conditional: 0.83 |
| LL + C_a_ - 6h-6h  (n = 48) | Intercept: 35.20 (p = 6.38e-11)  Slope: -8.29 (p = 0.0017) | [32.10; 38.31]  [-13.15; -3.43] | Variance = 0.82  Std. Dev. = 0.91 | Marginal: 0.19  Conditional: 0.20 |
| SPAD ~ (N - NO_3_^-^) | R equation: SPAD ~ (N – NO_3_^-^) + (1 \| PlantID) | | | |
| Experiment - treatment | Fixed effects | Fixed effects – 95% CI | Random effects | R² |
| HL + Ce + DY - 6h-6h  (n = 30) | Intercept: 4.80 (p = 0.021)  Slope: 0.045 (p = 2.07e-09) | [0.98; 8.61]  [0.035; 0.055] | Variance = 0.007  Std. Dev. = 0.084 | Marginal: 0.73  Conditional: 0.73 |

**
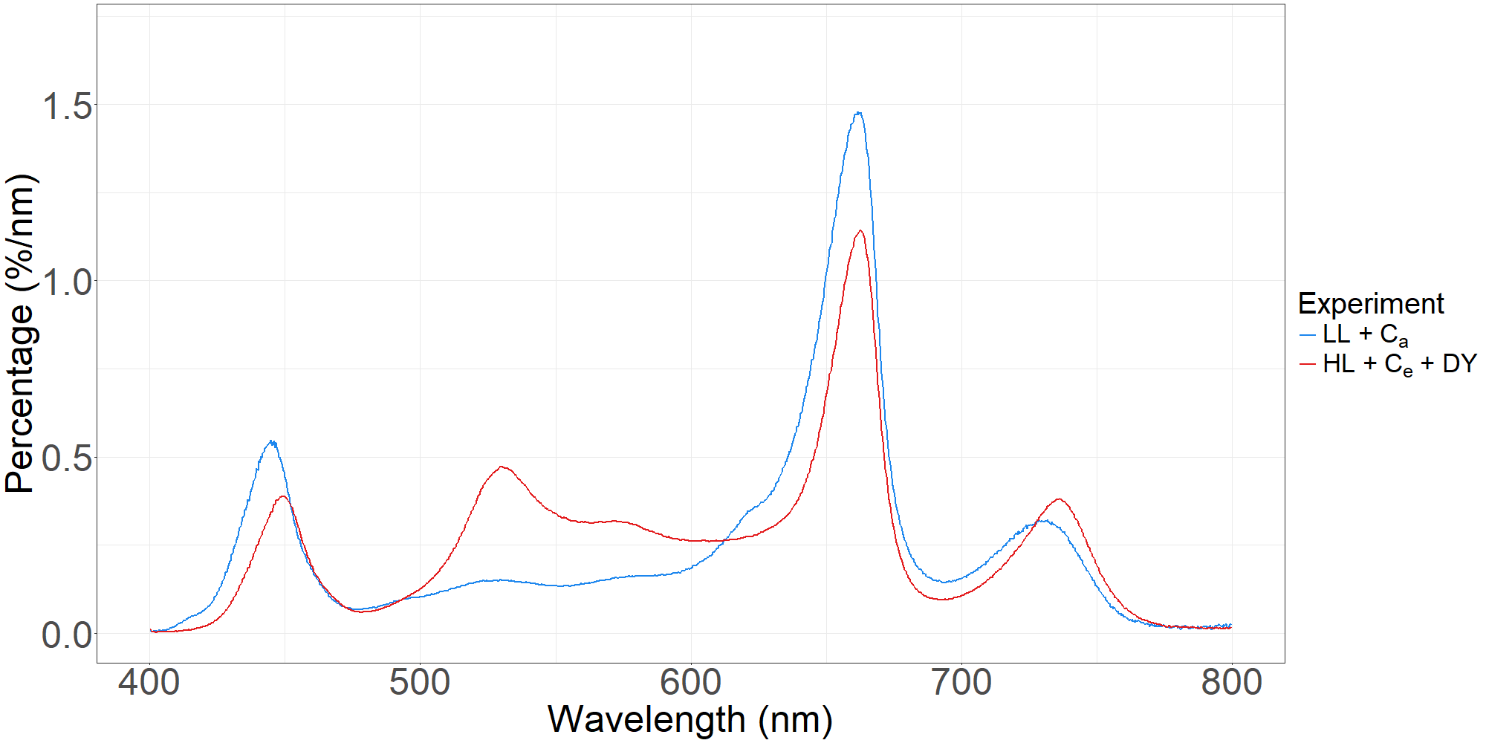
**

**Supplementary Figure S1.** The light spectra used in the low light and ambient CO_2_ concentration experiment (LL + C_a_) and the high light, elevated CO_2_ concentration with dynamic light fixtures experiment (HL + C_e_ + DY).


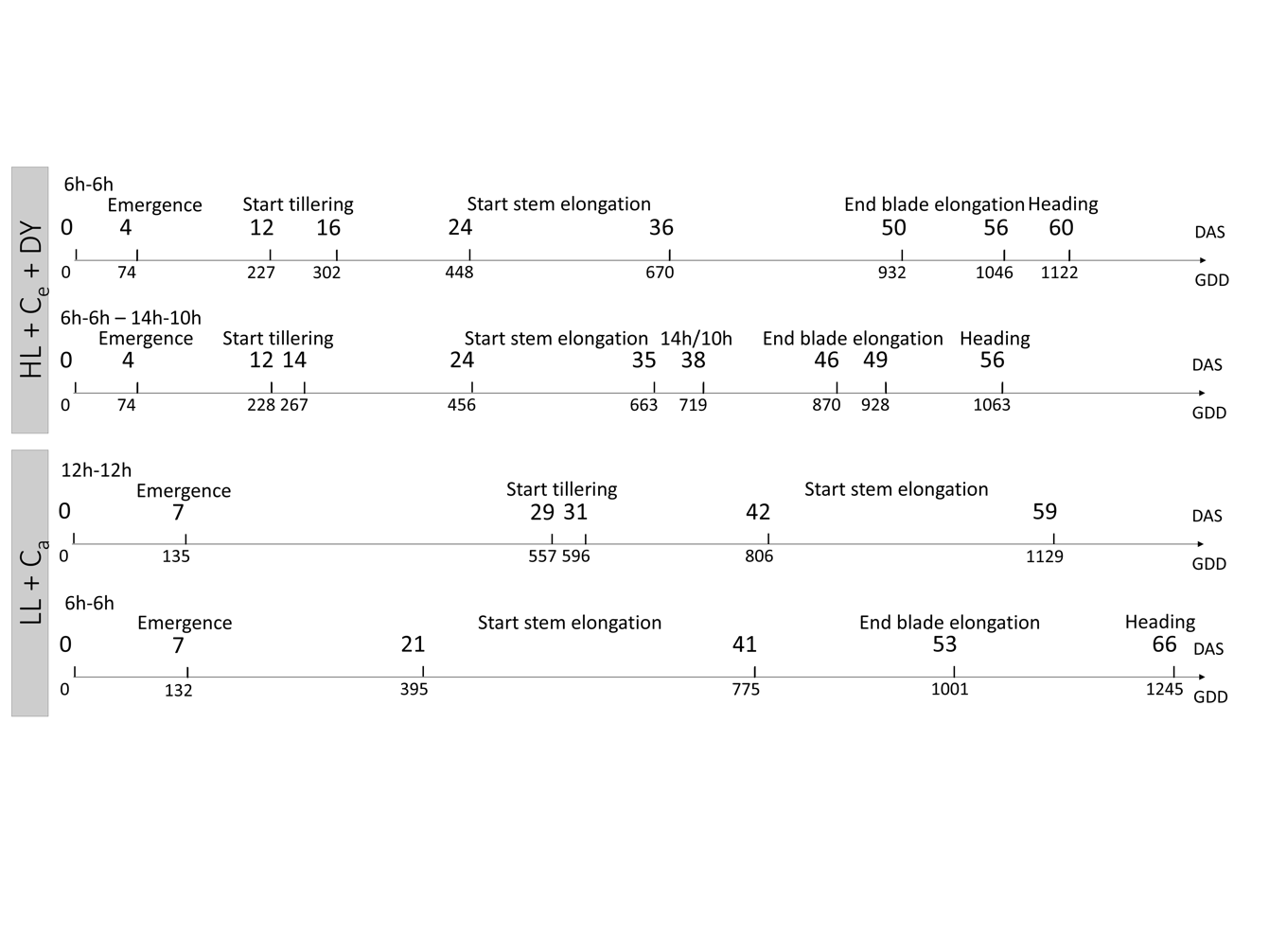
**Supplementary Figure S2.** Phenological development of the wheat plants in days after sowing (DAS) and thermal time in growing degree days (GDD, °Cd) in the HL + C_e_ + DY and LL + C_a_ experiments, for their respective light-dark cycles.


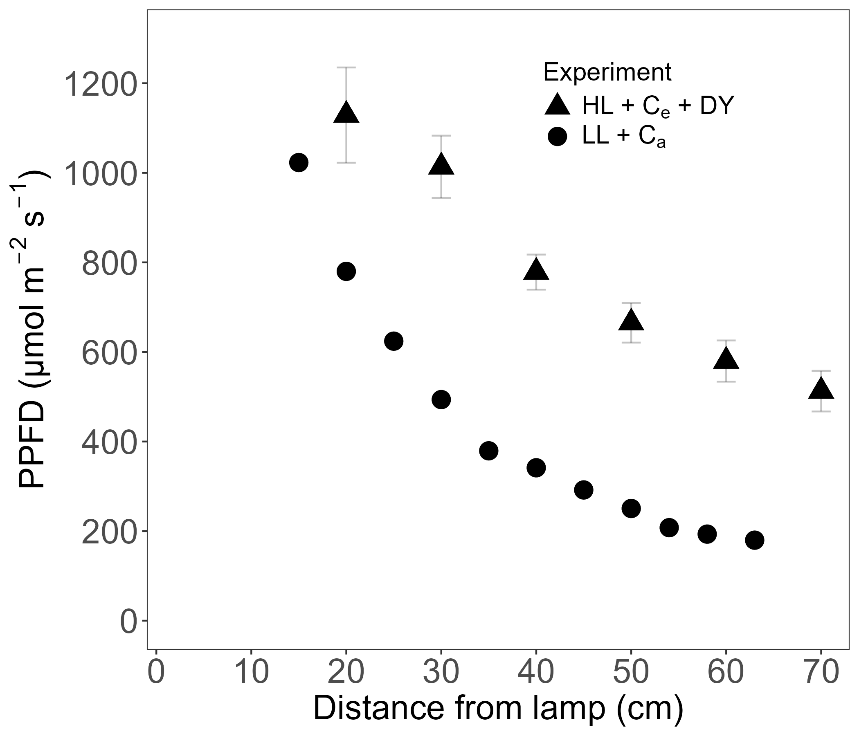


**Supplementary Figure S3.** Light intensity in function of the distance from the lamp in the HL + C_e_ + DY and LL + C_a_ experiments. Error bars indicate SD (n = 5 for HL + C_e_ + DY). Measurements of light intensity were repeated five times at each distance for HL + C_e_ + DY, and was measured once at each distance for LL + C_a_.


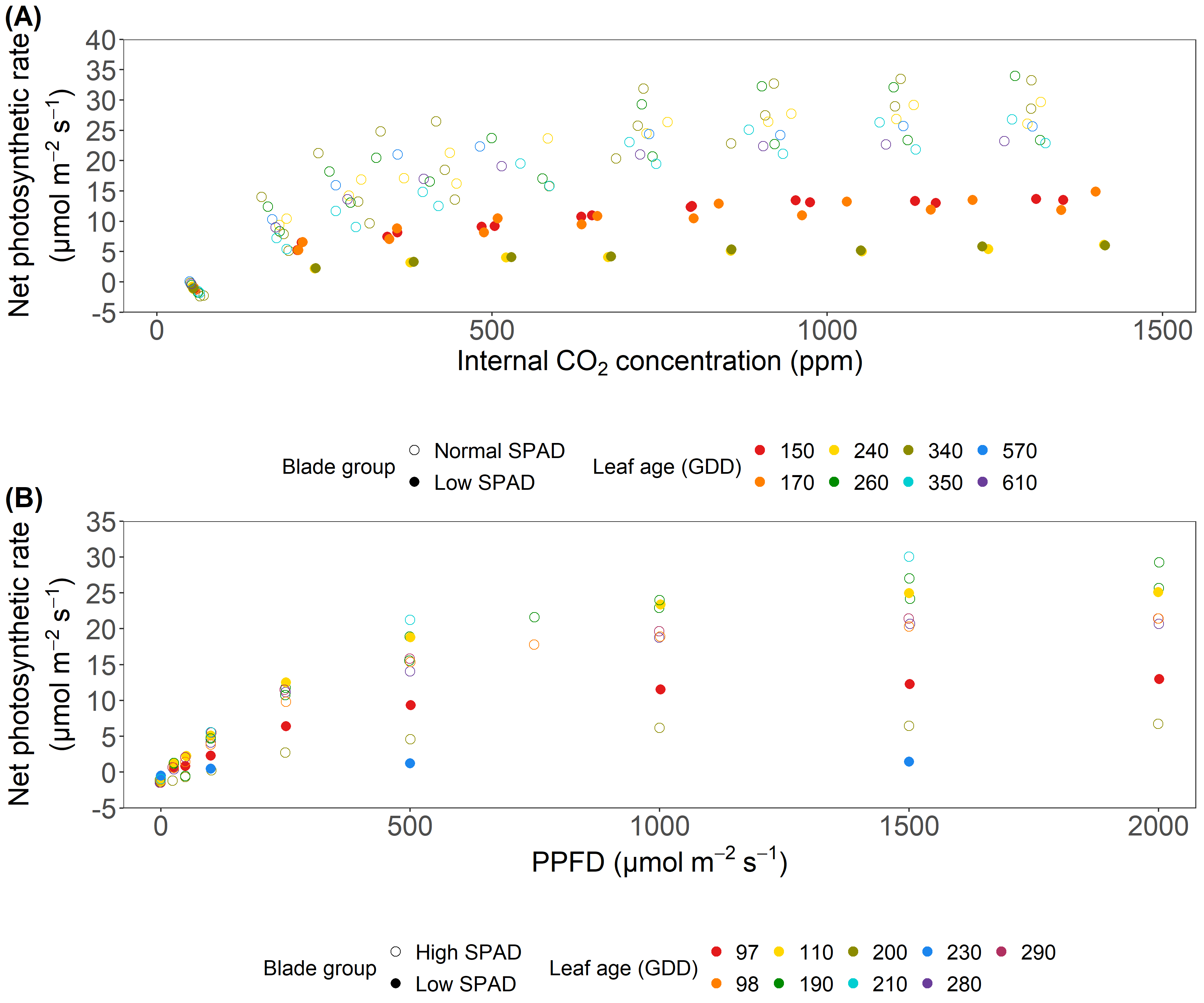


**Supplementary Figure S4.** CO_2_ response curves (AC_i_) and light response curves (LRC) for wheat blades sampled in the high light, elevated CO_2_ concentration with dynamic light fixtures experiment (HL + C_e_ + DY). **(A)** CO_2_ response curves showing blade net photosynthetic rate plotted against leaf internal CO_2_ concentration. **(B)** Light response curves showing blade net photosynthetic rate plotted against photosynthetic photon flux density (PPFD). Open circles indicate blades with a normal SPAD value (low nitrate content), closed circles indicate blades with a low-SPAD value (high nitrate content). Colors indicate leaf age. Only blades grown under a 6h-6h light-dark cycle are displayed.

**
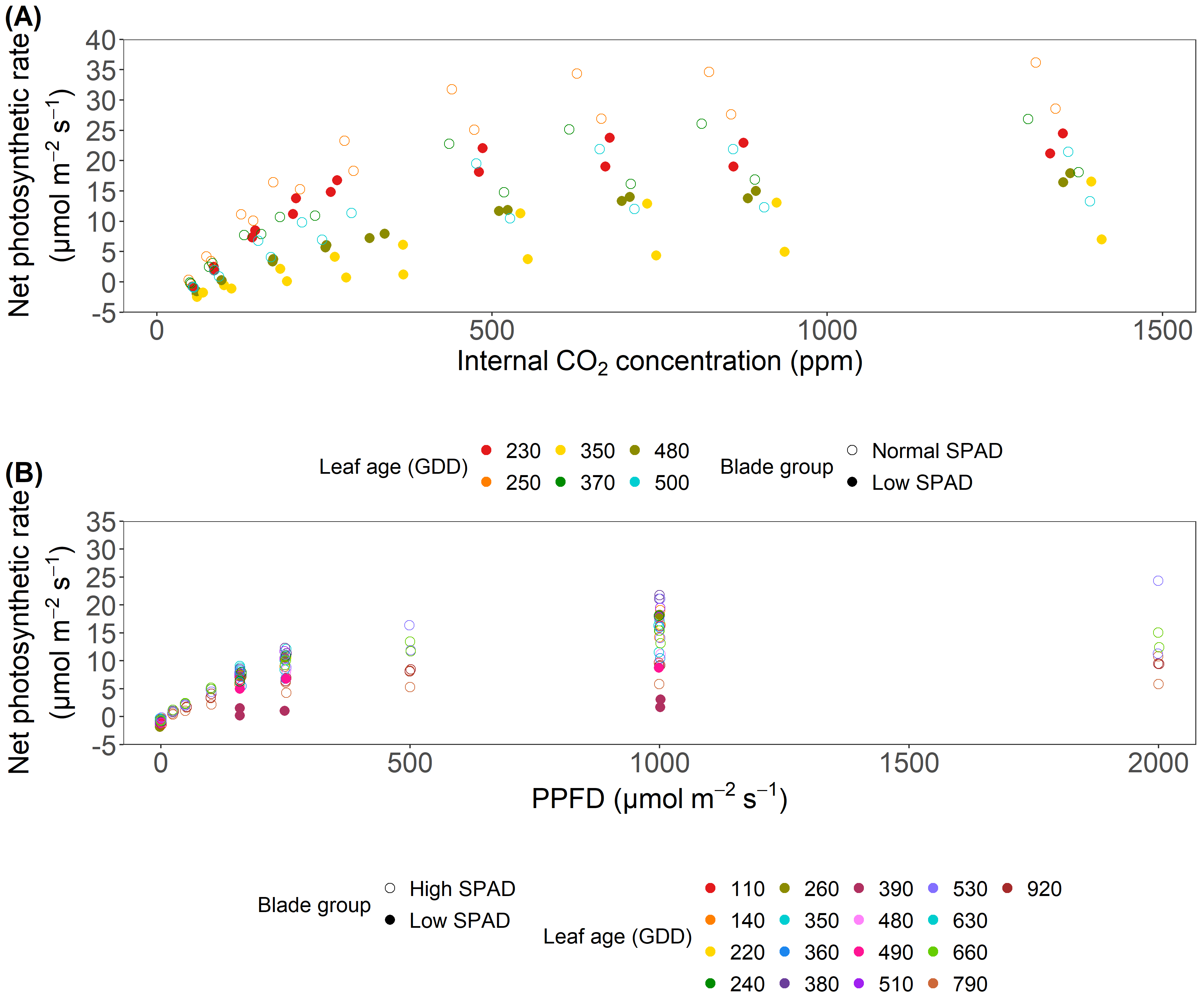
**

**Supplementary Figure S5.** CO_2_ response curves (AC_i_) and light response curves (LRC) for wheat blades sampled in the low light and ambient CO_2_ concentration experiment (LL + C_a_). **(A)** CO_2_ response curves showing blade net photosynthetic rate plotted against leaf internal CO_2_ concentration. **(B)** Light response curves showing blade net photosynthetic rate plotted against photosynthetic photon flux density (PPFD). Open circles indicate blades with a normal SPAD value (low nitrate content), closed circles indicate blades with a low-SPAD value (high nitrate content). Colors indicate leaf age. Only blades grown under a 6h-6h light-dark cycle are displayed.
